# Association of self-rated health with multimorbidity, chronic disease and psychosocial factors in a large middle-aged and older cohort from general practice: a cross-sectional study

**DOI:** 10.1186/s12875-014-0185-6

**Published:** 2014-11-25

**Authors:** Nahal Mavaddat, Jose M Valderas, Rianne van der Linde, Kay Tee Khaw, Ann Louise Kinmonth

**Affiliations:** Primary Care Unit, Department of Public Health and Primary Care, Strangeways Laboratory, 2 Worts Causeway, Cambridge, CB1 8RN UK; Health Services & Policy Research, University of Exeter Collaboration for Primary Care (APEx) and NIHR PenCLAHRC, University of Exeter Medical School, Smeall Building, St Luke’s Campus, Exeter, Devon EX1 2LU UK; Institute of Public Health, University of Cambridge, Forvie Site, Robinson Way, Cambridge, CB2 0SR UK; Clinical Gerontology Unit, Department of Public Health and Primary Care, Addenbrooke’s Hospital, Level 2, F+G Block, Box 251, Hills Road, Cambridge, CB2 2QQ UK; Primary Care Research Unit, University of Cambridge, Forvie Site, Robinson Way, Cambridge, CB2 0SR UK

**Keywords:** General practice/family medicine, General integrated subjective health multimorbidity comorbidity

## Abstract

**Background:**

The prevalence of coexisting chronic conditions (multimorbidity) is rising. Disease labels, however, give little information about impact on subjective health and personal illness experience. We aim to examine the strength of association of single and multimorbid physical chronic diseases with self-rated health in a middle-aged and older population in England, and to determine whether any association is mediated by depression and other psychosocial factors.

**Methods:**

25 268 individuals aged 39 to 79 years recruited from general practice registers in the European Prospective Investigation of Cancer (EPIC-Norfolk) study, completed a survey including self-rated health, psychosocial function and presence of common physical chronic conditions (cancer, stroke, heart attack, diabetes, asthma/bronchitis and arthritis). Logistic regression models determined odds of “moderate/poor” compared to “good/excellent” health by condition and number of conditions adjusting for psychosocial measures.

**Results:**

One-third (8252) reported one, around 7.5% (1899) two, and around 1% (194) three or more conditions. Odds of “moderate/poor” self-rated health worsened with increasing number of conditions (one (OR = 1.3(1.2–1.4)) versus three or more (OR = 3.4(2.3–5.1)), and were highest where there was comorbidity with stroke (OR = 8.7(4.6–16.7)) or heart attack (OR = 8.5(5.3–13.6)). Psychosocial measures did not explain the association between chronic diseases and multimorbidity with self-rated health.The relationship of multimorbidity with self-rated health was particularly strong in men compared to women (three or more conditions: men (OR = 5.2(3.0–8.9)), women OR = 2.1(1.1–3.9)).

**Conclusions:**

Self-rated health provides a simple, integrative patient-centred assessment for evaluation of illness in the context of multiple chronic disease diagnoses. Those registering in general practice in particular men with three or more diseases or those with cardiovascular comorbidities and with poorer self-rated health may warrant further assessment and intervention to improve their physical and subjective health.

## Background

As populations in developed countries age, so the number with single and multiple chronic conditions is increasing [1]. In recent international studies up to 50% of those with one chronic disease diagnosis have one or more other diagnoses; known as “multimorbidity”, approximating to 80% among those over 80 years [2-5]. Patients with multimorbidity suffer more, have poorer health outcomes including increased complications and earlier death, and require greater access to and use of primary and secondary health care than those with single conditions [6-12]. Tools are needed to enable integrative assessment of the impact of multimorbidity on patients so that services may be focussed according to individual need [13-15]. Available tools range from simple disease counts to standardized measures such as the Cumulative Illness Rating Scale (CIRS), the Charlson index (Charlson) and the Functional Comorbidity Index (FCI) [16-18]. While the latter are complex and designed for epidemiological rather than practice use, simple disease counts have shown to be valid and offer an intuitive approach to measurement [19-21]. For the clinic, however, a simple summary measure of individual health impact is needed which is easily measurable and reflects the illness experience of the patient with a number of chronic diseases.

Self-rated health (SRH) is elicited through a single question and could efficiently complement and individualise the count of common chronic diseases currently obtainable from General Practice records. It has been widely validated in epidemiological studies, reflects the subjective experience of health associated with more complex measures of health-related quality of life, and independently predicts health outcomes including all-cause mortality, disease specific mortality, morbidity and health service utilisation [22-26].

The presence of multimorbidity has been associated in previous studies with poor SRH [27-29]. However, what underlies this relationship has not been fully explored. For example, it is uncertain whether it may be predominantly psychological or social factors that mostly mediate the association of multimorbidity with SRH, or whether the association mostly reflects the physical dimensions of comorbid disease. The relationship between mental health, multimorbidity and SRH is complex. Mental health problems and psychosocial difficulties such as depression are often commonly comorbid with single or multiple chronic physical diseases [30-33]. The presence of poor psychosocial health in the context of chronic disease is also associated with worse subjective health assessment [31]. In a recent study, the comorbid state of depression with chronic physical diseases was associated with worse subjective health assessment than having depression alone or having any of a range of chronic diseases alone or in combination without depression [31]. The association of multimorbidity with depression may itself be mediated by a patients’ poor self-perceived health [30]. Previous work in the EPIC-Norfolk and other cohorts also confirms a strong relationship between SRH, mental health and social factors [34,35]. Multimorbidity particularly involving mental health disorders, is for example, especially increased in the context of social deprivation [32,33]. Further study is required to understand how multimorbidity and in particular specific disease combinations may impact on patients’ health experiences, and whether such experiences are mediated by modifiable psychosocial factors [19,20,36]. Identifying disease combinations leading to poorer SRH would also focus attention on those needing most help [37].

In this study, we aimed to explore the association between a number of common chronic physical conditions (heart disease, diabetes, arthritis, chronic lower respiratory tract disease and stroke) both as single and multimorbid conditions with SRH, assessing for the mediating effects of psychosocial function using data from a large well-characterised population derived from GP registers [38].

## Methods

### Study population

The study population comes from EPIC-Norfolk (Norfolk component of the European Prospective Investigation of Cancer) recruiting from general practice age-sex registers in Norwich city and surrounding towns and country (1993–1997) [39]. Ethical approval was obtained from the Norfolk Local Research Ethics Committee. Invitations were sent to all 39–79 year olds on the list of collaborating general practitioners. Over 30,000 of 77,630 (approximately 40%) of those approached returned a consent form indicating that they wished to join the study. Of these, 25 639 men and women aged 39–79 years attended a health examination, gave informed signed consent and completed a baseline detailed health and lifestyle questionnaire. Detailed descriptions of the study methodology have been reported [38]. This cohort is comparable to other national samples with respect to physical and psychosocial characteristics [38,40,41].

This cross-sectional analysis used both baseline data and additional psychological measures which were obtained at a later date by questionnaire (approximately 18 months later with a response rate of 17,268 (67%)). 371 individuals with missing data were excluded.

### Measures

The health questionnaire included the single-item SRH measure which asks: “In general, would you say your health is?” with response options “excellent”, “good”, “moderate” or “poor”.

Diagnoses of chronic medical conditions were determined by asking: “Has a doctor ever told you that you have any of the following?” followed by a list of options including cancer, stroke, heart attack, diabetes, respiratory disease (asthma or bronchitis) and arthritis. Self-reports of a previous diagnosis of depression as well as current antidepressant use (used as a proxy for current depression), were also elicited. Other psychosocial parameters were Emotional and Social Role Functioning subscales and Mental subscale of the Short-form SF-36 Health Survey [42], and short form of the Neuroticism scale of the Eysenck Personality Questionnaire (EPQ) [43], both validated questionnaires.

Social class was collected based on self-report and classified according to the Registrar General’s occupation based scheme into non-manual (I, II and III non-manual) and manual classes (III manual, IV and V) [44].

### Statistical analyses

Analyses used STATA statistical software version 11.0. The analysis is stratified by gender, since men and women may respond differently to the SRH question [45].

Descriptive statistics included means and percentages.

Differences by SRH categories in age, social class, chronic conditions and psychological measures were investigated. Two sample *t*-tests were used to compare differences in mean values between age categories, social class, chronic conditions and psychological measures. Assumption of equal variances was verified. Differences in percentages were compared using χ^2^-tests.

Univariate and multivariate logistic regressions models were constructed with SRH as a dependent variable to determine the odds of “moderate/poor” compared to “good/excellent” SRH for chronic conditions, psychosocial parameters, and for number of chronic conditions adjusting for age and social class.

Number of morbidities was calculated as a count of self-reported medical conditions: 0, 1, 2 and 3 or more. Multimorbidity was defined as the presence of two or more conditions. Since the focus of our research was the impact of physical disease on self-reported health status with mental health as a potential mediator, depression was not used in the count of conditions but as a covariate along with other measures of psychological and social functioning. Since any self-reported depression alone is also likely to be an underestimate of true psychological dysfunction in primary care patients, we also included separately in our model measures of psychosocial impairment mental health, emotional and social functioning and neuroticism, and antidepressant use. The 50th centile of scores for each of the Mental Health, Role Emotional and Social Functioning subscales of the SF-36, and the Neuroticism scale were determined and scores dichotomised into equal and above or below the median. Antidepressant use was dichotomized into yes or no.

A further co-morbidity model was constructed to determine the odds of “moderate/poor” compared to “good/excellent” SRH for each chronic condition as an index condition plus any one or more other conditions adjusted for age, social class depression and anti-depressant use.

## Results

Mean age was 59 years. 8252 (33%) reported one and 2093 (8%) more than one chronic condition. 20,101 (79.6%) rated their health as good or excellent and 5167 (20.4%) as moderate or poor (Table 1). 1,386 (5.5%) reported antidepressant use.

**Table 1:** **Demographic data, chronic conditions, number of conditions, psychological measures and self-rated health in EPIC-Norfolk**

	**Men**	**Women**	**Combined**
	**n = 11,439**	**n = 13,829**	**n = 25,268**
**Age** (yrs)	59.1 (9.3)	58.4 (9.3)	58.7 (9.3)
**Social Class**			
*Manual*	4670 (41.5)	5195 (38.5)	9865 (39.9)
*Non-manual*	6579 (58.5)	8290 (61.5)	14869 (60.1)
**Chronic conditions**			
*Cancer*	426 (3.7)	942 (6.8)	1368 (5.4)
*Stroke*	202 (1.8)	140 (1.0)	342 (1.4)
*Heart attack*	588 (5.1)	178 (1.3)	766 (3.0)
*Diabetes*	357 (3.1)	214 (1.5)	571 (2.3)
*Arthritis*	2619 (22.9)	3143 (22.7)	5762 (22.8)
*Respiratory (asthma or bronchitis)*	1681 (14.7)	2163 (15.6)	3844 (15.2)
**Number of reported conditions**			
*None*	6694 (58.5)	8229 (59.5)	14923 (59.1)
*One*	3739 (32.7)	4513 (32.6)	8252 (32.7)
*Two*	896 (7.8)	1003 (7.3)	1899 (7.5)
*Three or more*	110 (1.0)	84 (0.6)	194 (0.8)
**Self-rated health**			
*Excellent*	1981 (17.3)	2139 (15.5)	4120 (16.3)
*Good*	7146 (62.5)	8835 (63.9)	15981 (63.2)
*Moderate*	2093 (18.3)	2613 (18.9)	4706 (18.6)
*Poor*	219 (1.9)	242 (1.7)	461 (1.8)
**Psychological measures**			
*Self-reported depression%*	1509 (13.2)	1987 (14.4)	3496 (13.8)
*Antidepressant use%*	421 (3.7)	965 (7.0)	1,386 (5.5)
*SF-36 Mental health (0–100)*	79.0 (15.7)	75.8 (16.4)	77.2 (16.2)
*SF-36 Role emotional (0–100)*	85.5 (30.3)	82.2 (32.9)	83.7 (31.9)
*SF-36 Social functioning (0–100)*	87.3 (21.2)	85.8 (21.6)	86.5 (21.4)
*Neuroticism scale (0–12)*	3.7 (3.2)	4.9 (3.3)	4.4 (3.3)

(Data are n (%), except for age, and SF-36 and neuroticism scales (mean (SD))).

Frequency of “moderate/poor” SRH rose with greater age, lower social class, and indices of poor mental health across genders (Tables 2 and 3), but was not significantly different between the genders. Frequency of “moderate/poor” SRH also rose with the number of chronic conditions and was highest in those with stroke, heart attack and diabetes. However, even in these conditions, a minority reported excellent health. We tested the interaction between sex and multimorbidity in the association with SRH and found it to be significant (p-values of interaction term: one condition; 0.05, two conditions; <0.01, three or more conditions; <0.01), with men having a steeper rise in “moderate/poor” SRH with number of conditions, and being more likely to report “moderate/poor” SRH with stroke (p = .007).

**Table 2:** **Self-rated health by demographic data, chronic conditions and number of conditions in 11,439 men in EPIC-Norfolk**

	**Total**	**Excellent**	**Good**	**Moderate**	**Poor**
**Age**					
*39–64*	7666 (67.0)	1444 (18.8)	4796 (62.6)	1278 (16.7)	148 (1.9)
*>65*	3773 (33.0)	537 (14.2)	2350 (62.3)	815 (21.6)	71 (1.9)
**Social class**					
*Manual*	4670 (41.5)	584 (12.5)	2872 (61.5)	1096 (23.5)	118 (2.5)
*Non-manual*	6579 (58.5)	1359 (20.7)	4168 (63.4)	957 (14.5)	95 (1.4)
**Chronic conditions**					
*Cancer*	426 (3.7)	48 (11.3)	260 (61.0)	104 (24.4)	14 (3.3)
*Stroke*	202 (1.8)	6 (3.0)	84 (41.6)	96 (47.5)	16 (7.9)
*Heart attack*	588 (5.1)	29 (4.9)	261 (44.4)	245 (41.7)	53 (9.0)
*Diabetes*	357 (3.1)	11 (3.1)	190 (53.2)	128 (35.9)	28 (7.8)
*Arthritis*	2619 (22.9)	430 (16.4)	1598 (61.0)	534 (20.4)	57 (2.2)
*Respiratory (asthma or bronchitis)*	1681 (14.7)	291 (17.3)	1068 (63.5)	293 (17.4)	29 (1.7)
**Number of conditions**					
*None*	6694 (58.5)	1278 (19.1)	4289 (64.1)	1033 (15.4)	94 (1.4)
*One*	3739 (32.7)	593 (15.6)	2302 (61.6)	771 (20.6)	73 (2.0)
*Two*	896 (7.8)	108 (12.1)	509 (56.8)	244 (27.2)	35 (3.9)
*Three or more*	110 (1.0)	2 (1.8)	46 (41.8)	45 (40.9)	17 (15.5)
**Psychological measures**					
*Self-reported depression%*	1509 (13.2)	260 (17.2)	932 (61.8)	287 (19.0)	30 (2.0)
*Antidepressant use%*	421 (3.7)	28 (6.7)	203 (48.2)	155 (36.8)	35 (8.3)
*SF-36 Mental health(0–100)*	79.0 (15.7)	84.6 (12.6)	80.2 (14.6)	70.2 (17.6)	62.9 (20.4)
*SF-36 Role emotional(0–100*	85.5 (30.3)	93.2 (20.4)	87.9 (27.6)	71.3 (39.8)	54.5 (45.4)
*SF-36 Social functioning(0–100)*	87.3 (21.2)	94.3 (14.4)	89.8 (18.5)	74.3 (26.0)	51.1 (32.7)
*Neuroticism scale (0–12)*	3.7 (3.2)	2.9 (2.7)	3.6 (3.1)	5.0 (3.4)	5.4 (3.6)

(Data are all n(%), except for SF-36 and neuroticism scales (mean (SD)).

**Table 3:** **Self-rated health by demographic data, chronic conditions and number of conditions in 13,829 women in EPIC-Norfolk**

	**Total**	**Excellent**	**Good**	**Moderate**	**Poor**
**Age**					
*39–64*	9642 (69.7)	1631 (16.9)	6215 (64.5)	1647 (17.1)	149 (1.5)
*>65*	4187 (30.3)	508 (12.1)	2620 (62.6)	966 (23.1)	93 (2.2
**Social class**					
*Manual*	5195 (38.5)	607 (11.7)	3271 (63.0)	1198 (23.1)	119 (2.3)
*Non-manual*	8290 (61.5)	1477 (17.8)	5371 (64.8)	1329 (16.0)	113 (1.4)
**Chronic conditions**					
*Cancer*	942 (6.8)	94 (10.0)	550 (58.4)	261 (27.7)	37 (3.9)
*Stroke*	140 (1.0)	9 (6.4)	73 (52.1)	45 (32.1)	13 (9.3)
*Heart attack*	178 (1.3)	5 (2.8)	77 (43.3)	76 (42.7)	20 (11.2)
*Diabetes*	214 (1.5)	8 (3.7)	111 (51.9)	79 (36.9)	16 (7.5)
*Arthritis*	3143 (22.7)	474 (15.1)	2015 (64.1)	608 (19.3)	46 (1.5)
*Respiratory(asthma or bronchitis)*	2163 (15.6)	312 (14.4)	1399 (64.7)	412 (19.0)	40 (1.8)
**Number of conditions**					
*None*	8229 (59.5)	1370 (16.6)	5322 (64.7)	1420 (17.3)	117 (1.4)
*One*	4513 (32.6)	642 (14.2)	2852 (63.2)	932 (20.7)	87 (1.9)
*Two*	1003 (7.3)	121 (12.1)	615 (61.3)	236 (23.5)	31 (3.1)
*Three or more*	84 (0.6)	6 (7.1)	46 (54.8)	25 (29.8)	7 (8.3)
**Psychological measures**					
*Self-reported depression%*	1987 (14.4)	336 (16.9)	1280 (64.4)	337 (17.0)	34 (1.7)
*Antidepressant use%*	965 (7.0)	57 (5.9)	511 (53.0)	336 (34.8)	61 (6.3)
*SF-36 Mental health (0–100)*	75.8 (16.4)	82.3 (13.7)	76.6 (15.4)	67.4 (17.8)	59.9 (28.1)
*SF-36 Role emotional (0–100)*	82.2 (32.9)	90.9 (23.3)	84.3 (30.9)	67.6 (40.9)	51.5 (45.6)
*SF-36 Social functioning (0–100)*	85.8 (21.6)	93.4 (15.2)	88.0 (19.4)	73.2 (26.0)	47.1 (28.1)
*Neuroticism scale (0–12)*	4.9 (3.3)	3.7 (2.9)	4.8 (3.2)	6.2 (3.3)	6.4 (3.5)

(Data are all n(%), except for SF-36 and neuroticism scales (mean (SD)).

Table 4 demonstrates the relationships between psychosocial measures, chronic disease and SRH. In men, poor scores on the SF-36 Social Functioning scale were more strongly associated with “moderate/poor” SRH than having a heart attack; and SF-36 Mental Health scores and antidepressant use than with having a stroke or diabetes. In women, SF-36 Social Functioning and Mental Health and antidepressant use were more strongly associated with SRH than having a stroke or diabetes but not with having had a heart attack. Adjustment for psychosocial measures reduced the odds of “moderate/poor” SRH in those with stroke. Odds of reporting “moderate/poor” SRH increased with the number of conditions in both men and women, but the strength of the association was greater for men (2 conditions: OR 2.1 (1.8-2.5); 3 or more conditions: OR 5.8 (3.9-8.5)) than for women (2 conditions: OR 1.5 (1.3-1.8); 3 or more conditions: OR 2.2 (1.4-3.6)). Adjustment for psychosocial measures did not significantly reduce the odds of poor SRH in those with multimorbid conditions.

**Table 4:** **Logistic regression models for “moderate/poor” compared to “good/excellent” SRH for chronic conditions and psychosocial measures in EPIC-Norfolk**

	**Men OR (95% CI)**	**Women OR (95% CI)**	Combined* OR (95% CI)
**Age**	**(N = 11,439)**	**(N = 13,829)**	**(N = 25268)**
*>65 vs 39–64*	1.3 (1.2–1.5)	1.5 (1.4–1.6)	1.4 (1.3–1.5)
**Social Class**	**(N = 11,249)**	**(N = 13,485)**	**(N = 24734)**
*Non-manual vs manual*	0.5 (0.5–0.6)	0.6 (0.6–0.7)	0.6 (0.5–0.6)
***Sex***			**(N = 25268)**
*Female vs male*	-	-	1.0 (1.0–1.1)
**Psychosocial measures**	**(N = 7619)**	**(N = 9676)**	**(N = 17295)**
*Diagnosis of depression (present vs absent)* ^**†**^	1.1 (0.9–1.2)	0.8 (0.8–1.0)	0.9 (0.9–1.0)
*Antidepressant use (use vs no use)* ^**†**^	3.7 (3.0–4.5)	2.9 (2.6–3.4)	3.2 (2.8–3.5)
*SF-36 Mental health (>80 vs. ≤80 )* ^**†**^	3.7 (3.2–4.2)	3.0 (2.7–3.4)	3.3 (3.0–3.6)
*SF-36 Role Emotional (=100 vs. ≤99)* ^**†**^	3.1 (2.7–3.5)	2.8 (2.5–3.1)	2.9 (2.7–3.1)
*SF-36 Social Functioning (=100 vs. ≤99)* ^**†**^	4.6 (4.1–5.5)	4.0 (3.5–4.4)	4.2 (3.9–4.6)
*Neuroticism scale (<4 vs > =4)* ^**†**^	2.5 (2.2–2.8)	2.4 (2.1–2.6)	2.4 (2.2–2.6)
**Chronic conditions**	**(N = 11,439)**	**(N = 13,829)**	**(N = 25268)**
Cancer^**†**^	1.5 (1.2–1.8)	1.8 (1.6–2.1)	1.7 (1.5–1.9)
*Cancer adjusted* ^‡^	*1.3 (0.9–1.0)*	*1.8 (1.4–2.2)*	*1.6 (1.4–1.9)*
Stroke^**†**^	4.5 (3.3–6.0)	2.4 (1.7–3.3)	3.4 (2.7–4.3)
*Stroke adjusted* ^‡^	*3.1 (2.1–4.7)*	1.6 (0.9–2.7)**	*2.3 (1.7–3.2)*
Heart attack^**†**^	4.1 (3.5–4.9)	3.8 (2.8–5.2)	4.0 (3.4–4.6)
*Heart attack adjusted* ^‡^	*4.2 (3.3–5.3)*	*4.3 (2.9–6.5)*	*4.1 (3.4–5.1)*
Diabetes^**†**^	2.9 (2.3–3.6)	2.9 (2.2–3.8)	2.9 (2.4–3.4)
*Diabetes adjusted* ^‡^	*2.5 (1.9–3.4)*	*2.5 (1.7–3.7)*	*2.5 (2.0–3.2)*
Arthritis^**†**^	1.2 (1.1–1.3)	1.0 (1.0–1.1)	1.1 (1.0–1.2)
*Arthritis adjusted* ^‡^	1.1 (1.0–1.3)**	1.0 (0.8–1.1)**	1.0 (0.9–1.1)**
Respiratory (asthma or bronchitis)^**†**^	0.9 (0.8–1.1)	1.1 (0.9–1.2)	1.0 (0.9–1.1)
*Respiratory adjusted* ^‡^	0.9 (0.8–1.1)**	*1.2 (1.0–1.3)*	1.1 (0.9–1.2)**
**Multimorbidity**	**(N = 11,439)**	**(N = 13,829)**	**(N = 25268)**
None reported^**†**^	Ref	Ref	Ref
*None adjusted* ^‡^	Ref	Ref	Ref
One^**†**^	1.4 (1.3–1.6)	1.3 (1.2–1.4)	1.3 (1.2–1.4)
*One adjusted* ^‡^	*1.4 (1.2–1.6)*	*1.3 (1.1–1.4)*	*1.3 (1.2–1.4)*
Two conditions^**†**^	2.1 (1.8–2.5)	1.5 (1.3–1.8)	1.8 (1.6–2.0)
*Two adjusted* ^‡^	*1.9 (1.5–2.3)*	*1.6 (1.3–2.0)*	*1.7 (1.5–2.0)*
Three or more conditions^**†**^	5.8 (3.9–8.5)	2.2 (1.4–3.6)	3.8 (2.8–5.1)
*Three adjusted* ^‡^	*5.2 (3.0–8.9)*	*2.1 (1.1–3.9)*	*3.4 (2.3–5.1)*

^**†**^Adjusted for age and social class ^‡^adjusted for age and social class and psychosocial measures (depression, antidepressant use, SF-36 mental and social functioning, neuroticism scale), *also adjusted for sex. All adjusted OR’s significant at p<.05 except those marked**.

**Figure 1:**
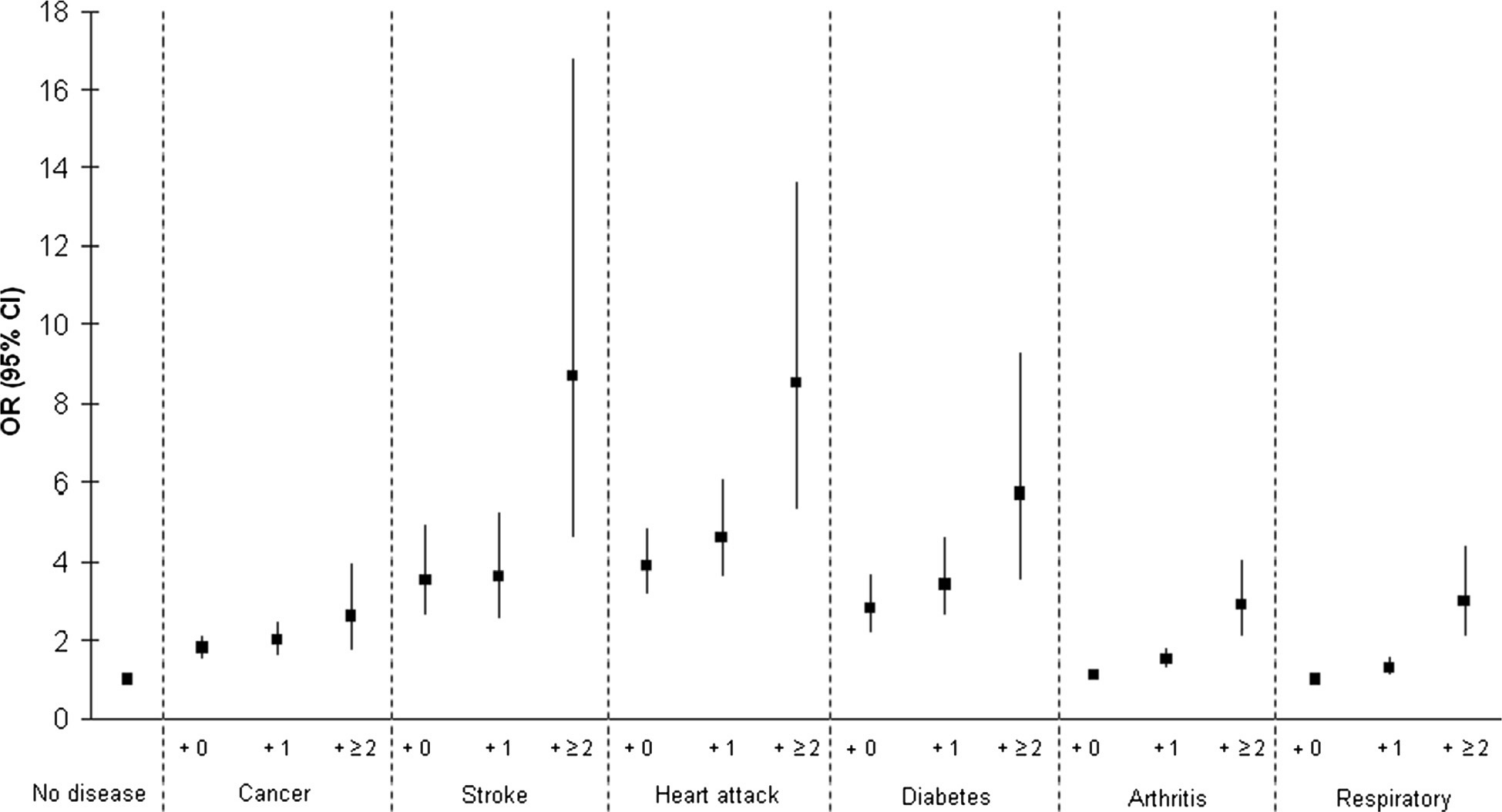
**Univariate effect of specific disease combinations versus no disease on “poor” compared to “good” SRH.** Logistic Regression Models: Univariate effect of specific disease combinations* versus no disease on “poor” SRH compared to “good” SRH adjusted for age sex and social class and antidepressant use in 11,439 men and 13,829 women in EPIC-Norfolk.

The odds of reporting “moderate/poor” SRH in those with two or more conditions in addition to any index condition was also roughly twice that of reporting only one other condition, even among the less common conditions where confidence intervals were wider (Table 5, Figure 1). Combinations with stroke and two or more conditions in men had the highest odds of “moderate/poor” SRH (OR 13.0 (5.4–31.1)), followed by combinations of two or more conditions with heart attack (OR 10.3(5.9–18.0)) and with diabetes (OR 9.4 (5.0–17.6)). The relationship between SRH and combinations of conditions was not significantly altered by correcting for self-reported depression and current antidepressant use.

**Table 5:** **Univariate logisitic regression models for “moderate/poor” compared to “good/excellent” SRH for disease combinations in EPIC-Norfolk**

	**Men**		**Women**		Combined *	
**Disease combinations**	**N**	**OR (95% CI) Ref = no disease**	**N**	**OR (95% CI) Ref = no disease**	**N**	**OR (95% CI) Ref = no disease**
**Cancer**						
Cancer only ^**†**^	249	1.5 (1.1–2.1)	578	1.9 (1.6–2.3)	827	1.8 (1.5–2.1)
*Cancer adjusted* ^‡^		1.5 (1.1–2.1)		*1.9 (1.6–2.3)*		*1.8 (1.5–2.1)*
Cancer + one condition ^**†**^	132	1.8 (1.2–2.6)	309	2.1 (1.6–2.7)	441	2.0 (1.6–2.4)
*Cancer + one adjusted* ^‡^		1.8 (1.2–2.6)		*2.0 (1.6–2.6)*		*2.0 (1.6–2.4)*
Cancer + two or more conditions ^**†**^	45	3.9 (2.1–7.1)	55	1.8 (1.0–3.3)	100	2.6 (1.7–3.9)
*Cancer + two adjusted* ^‡^		3.9 (2.1–7.2)		*1.8 (1.0–3.3)*		*2.6 (1.7–3.9)*
**Stroke**						
Stroke only ^**†**^	101	4.3 (2.8–6.5)	70	2.9 (1.8–4.8)	171	3.6 (2.6–5.0)
*Stroke adjusted* ^‡^		*4.2 (2.8–6.3)*		*2.9 (1.7–4.8)*		*3.5 (2.6–4.9)*
Stroke + one condition ^**†**^	73	5.4 (3.4–8.8)	54	2.1 (1.2–3.7)	127	3.6 (2.5–5.2)
*Stroke + one condition adjusted* ^‡^		*5.4 (3.3–8.8)*		*2.0 (1.1–3.7)*		*3.6 (2.5–5.2)*
Stroke + two or more conditions ^**†**^	28	13.1 (5.5–31.1)	16	4.3 (1.5–11.9)	44	8.4 (4.4–16.1)
*Stroke + two or more conditions adjusted* ^‡^		*13.0 (5.4–31.1)*		*4.8 (1.7–13.3)*		*8.7 (4.6–16.7)*
**Heart attack**						
Heart attack only ^**†**^	319	4.1 (3.2–5.2)	96	3.5 (2.3–5.3)	415	3.9 (3.2–4.8)
*Heart attack only adjusted* ^‡^		*4.1 (3.2–5.2)*		*3.5 (2.3–5.4)*		*3.9 (3.2–4.8)*
Heart attack + one condition ^**†**^	206	4.5 (3.3–6.0)	62	5.8 (3.4–10.0)	268	4.6 (3.6–-6.0)
*Heart attack + one condition adjusted* ^‡^		*4.4 (3.3–5.9)*		*6.0 (3.5–10.4)*		*4.6 (3.6–6.0)*
Heart attack + two or more conditions ^**†**^	63	10.3 (5.9–17.8)	20	4.4 (1.7–11.4)	83	8.3 (5.2–13.3)
*Heart attack + two or more conditions adjusted* ^‡^		*10.3 (5.9–18.0)*		*4.7 (1.8–12.2)*		*8.5 (5.3–13.6)*
**Diabetes**						
Diabetes only ^**†**^	177	2.5 (1.8–3.4)	112	3.4 (2.3–5.0)	289	2.8 (2.2–3.6)
*Diabetes only adjusted* ^‡^		*2.5 (1.8–3.4)*		*3.4 (2.0–5.1)*		*2.8 (2.2–3.6)*
Diabetes + one condition ^**†**^	134	3.7 (2.6–5.3)	75	3.1 (2.0–5.0)	209	3.5 (2.6–4.6)
*Diabetes + one condition adjusted* ^‡^		*3.6 (2.5–5.2)*		*3.2 (2.0–5.1)*		*3.4 (2.6–4.6)*
Diabetes + two or more conditions ^**†**^	46	9.3 (5.0–17.4)	27	2.8 (1.3–6.3)	73	5.9 (3.7–9.5)
*Diabetes + two or more conditions adjusted* ^‡^		*9.4 (5.0–17.6)*		*2.4 (1.1–5.4)*		*5.7 (3.5–9.2)*
**Arthritis**						
Arthritis only ^**†**^	1,854	1.2 (1.0–1.3)	2,265	1.1 (0.9–1.2)	4119	1.1 (1.0–1.2)
*Arthritis only adjusted* ^‡^		*1.2 (1.0–1.4)*		1.1 (0.9–1.2)**		*1.1 (1.0–1.2)*
Arthritis + one condition ^**†**^	678	1.8 (1.5–2.2)	805	1.3 (1.1–1.5)	1483	1.5 (1.3–1.7)
*Arthritis + one condition adjusted* ^‡^		*1.8 (1.5–2.2)*		*1.3 (1.1–1.5)*		*1.5 (1.3–1.7)*
Arthritis + two or more conditions ^**†**^	87	4.6 (3.0–7.2)	73	1.7 (1.0–2.9)	160	3.0 (2.1–4.1)
*Arthritis + two or more conditions adjusted* ^‡^		*4.7 (3.0–7.2)*		*1.6 (1.0–2.7)*		*2.9 (2.1–4.0)*
**Respiratory (asthma or bronchitis)**						
Respiratory only ^**†**^	1,039	0.9 (0.7–1.1)	1,392	1.1 (0.9–1.2)	2431	1.0 (0.9–1.1)
*Respiratory only adjusted* ^‡^		0.9 (0.7–1.1)**		1.1 (0.9–1.3)**		1.0 (0.9–1.1)**
Respiratory + one condition ^**†**^	569	1.4 (1.1–1.7)	701	1.2 (1.0–1.5)	1270	1.3 (1.1–1.5)
*Respiratory + one condition adjusted* ^‡^		*1.4 (1.1–1.7)*		*1.2 (1.0–1.5)*		*1.3 (1.1–1.5)*
Respiratory + two or more conditions ^**†**^	73	4.0 (2.5–6.5)	70	2.2 (1.3–3.7)	143	3.0 (2.2–4.3)
*Respiratory + two or more conditions adjusted* ^‡^		*4.2 (2.6–6.7)*		*2.1 (1.3–3.6)*		*3.0 (2.1–4.3)*

^**†**^Adjusted for age,social class ^‡^adjusted for age, social class and antidepressant use *adjusted also for sex. All adjusted OR’s significant at p<.05 except those marked**.

## Discussion

### Summary of main findings

In this large middle-aged to older population, poorer subjective health in the form of SRH is strongly associated with single chronic physical conditions (particularly cardiovascular diseases: heart attack, stroke and diabetes), and multiple morbidities (particularly three or more chronic conditions (OR 5.2 (3.0–8.9)). Odds of poorer SRH also rose especially in the presence of cardiovascular diseases particularly where there was comorbidity with stroke or heart attack. Men with three or more conditions had nearly two and a half times the risk of poorer SRH than women, with the increase risk especially with multimorbidity involving stroke, heart attack or diabetes.

A strong association was present between poor SRH and poor psychosocial function, as shown in previous studies [46,47]. In particular SRH has been shown to be strongly associated with SF-36 mental health and social functioning measures [48]. However, adjusting for psychosocial measures did not significantly modify the association between chronic conditions, multimorbidity and SRH in this study. Only among those with stroke, was there a slight weakening of the association with SRH after controlling for psychological and social functioning. This may relate in part to the confounding effects of poor cognitive function on psychosocial measures in this population, which was not evaluated in this study. Our findings, therefore, suggest that while psychological dysfunction and social deprivation and functioning are an important aspect of the multimorbidity burden, they do not fully explain the association between physical multimorbidity and poorer subjective health. The relationship between overall multimorbidity burden and subjective health is likely to be determined by a complex interaction of factors including physiological interactions between diseases or the drugs used in their management, and the behavioural burden of monitoring and treatment regimes and self-management [19,29,30,49].

Other studies have assessed the association of particular combinations of chronic conditions with SRH [37]. In our study, analyses of the association of increasing numbers of conditions added to a single index condition helped identify those chronic conditions most likely to contribute to morbidity burden in association with other conditions. Our analysis included only combinations of significant common chronic physical conditions. However, it suggests that particular combinations such as those involving the cardiovascular system may potentially lead to the greatest comorbidity burdens, independent of the presence of psychosocial factors. The relationships between disease burden and SRH especially in cases of multimorbidity involving cardiovascular diseases were greater in men than in women. Addressing poorer SRH in those with cardiovascular disease in combination with other conditions may be particularly important, since poor SRH has previously been shown to be strongly associated with poor cardiovascular outcomes [25].

### Strengths and limitations

Strengths of this study include a large well-characterized General Practice based population comparable to other national samples in respect to both physical and psychosocial characteristics [38,40,41]. Functional status, in particular, is comparable to that documented for similar age groups in two UK studies: The ‘Health Survey for England’ and the ‘Omnibus Survey in Great Britain’ - although the physical component summary scores in EPIC-Norfolk are slightly lower compared to the Oxford Health Life Survey [38,40,41]. Other strengths are that common chronic diseases were studied, all except arthritis being included in the English Quality Outcomes Framework (QOF) [50]. Not all possible chronic conditions, however, were included in the count of morbidities. Our study reports an approximately 8% rate of multimorbidity in this GP-derived population. Based on a recent systematic review by Violan et al. (2014), this is lower than the lowest rate of multimorbidity reported in any population (12.5% in a Dutch primary care cohort) [5,51]. The reasons for this may include the use of only five conditions in the count of multimorbidity, self-report of conditions rather than reports derived from medical records, and the fact that the oldest old were not included in the study [52]. Self-report of doctor-confirmed diagnoses were used in this study and may be more sensitive to identifying symptoms-based conditions [53], but may also lead to recall bias; for example those with worse SRH may recall more diagnoses. The severity and duration of conditions were not elicited in this study. Reports were of having ever been diagnosed with the condition, and therefore some conditions may have not been currently limiting. Thus diagnosis of depression which was not related to poorer subjective health, or cancer which was weakly related, may be due to past rather than current disease or ‘healthy survivor’ effect in the case of cancer. Indeed current anti-depressant use showed stronger association with poorer SRH than ever-diagnosis of depression. The time gap between psychological measures and the remainder of the questions elicited in the study may have reduced the association of psychological measures with SRH. Socioeconomic measures in the EPIC-Norfolk were also limited to social class and education. Time spent on health related activities is increased in the presence of multimorbidity and may well mediate the association with SRH, but no information on this was available in our study [54]. Finally, the cross-sectional design limits interpretation and poorer subjective health may itself predict onset and severity of multimorbidity.

### Implications of findings

This study has implications for both primary care practice and future research. Primary care is central to providing patient-centred care for patients with chronic illnesses and multimorbidity. Tools for enabling care of patients with multiple chronic conditions already include multimorbidity registers, chronic condition clinics, guidelines for management of comorbid conditions, monitoring coordination of care, and improving self-care and patient education [49,55]. Calls have also been made to screen for depression for those with multiple chronic conditions in primary care [56]. Similarly, SRH elicited from patients at registration or review, at the beginning of consultations verbally or in written form would contribute to these tools. Eliciting SRH, especially among those with a history of three or more chronic diseases (in particular men) may help to identify a group at risk of both poorer current function and premature mortality, in particular when the diagnosis involves a history of cardiovascular disease. A recent study has shown improved outcomes including health-related quality of life with a chronic disease self-management programme, most frequently in those who had multiple physical conditions plus ‘probable depression’ [56]. A similar approach could aim interventions at those with multimorbidity who report poorer SRH. Such interventions should focus on physical as well as mental aspects of improving health status in patients with multimorbidity.

The findings of this study raise questions for further research especially the extent to which SRH fulfills the criteria for screening for burden of illness in multimorbid states; longitudinal study is needed to confirm the relationship between SRH and multimorbidity with better characterisation of disease severity and psychological vulnerability. Development of studies to explore in greater detail the subjective health of those with three or more chronic conditions, specific combinations of conditions associated with greatest illness burden, and to elicit further information on what lies behind the significant rise in subjective poorer health as numbers of conditions increase would be of value. Exploration of discordant findings such as factors associated with excellent SRH in the face of multiple diagnoses may also be fruitful.

## Conclusions

In conclusion, the reporting of poorer self-rated health rises significantly in those with three or more chronic conditions. Self-rated health provides an integrative patient-centred assessment of multimorbidity burden, over and above psychosocial measures, and should be used in the evaluation and planning of care of patients with multimorbidity in general practice. Further research into the use of self-rated health as a screening tool in general practice is required.
